# Methodological comparisons for antimicrobial resistance surveillance in feedlot cattle

**DOI:** 10.1186/1746-6148-9-216

**Published:** 2013-10-21

**Authors:** Katharine M Benedict, Sheryl P Gow, Sylvia Checkley, Calvin W Booker, Tim A McAllister, Paul S Morley

**Affiliations:** 1 Department of Clinical Sciences, Colorado State University, Fort Collins, CO 80523-1678, USA; 2 Laboratory for Foodborne Zoonoses, Public Health Agency of Canada, University of Saskatchewan, Saskatoon, SK, S7N 5B4, Canada; 3 Faculty of Veterinary Medicine, University of Calgary, Calgary, AB T2N 4Z6, Canada; 4 Feedlot Health Management Services Ltd, Okotoks, AB, T1S 2A2, Canada; 5 Lethbridge Research Center, University of Lethbridge, Lethbridge, AB T1J 4B1, Canada

**Keywords:** Antibiotic resistance, Cattle, Escherichia coli, Mannheimia haemolytica, Susceptibility testing, Broth microdilution, Disk diffusion, Sampling

## Abstract

**Background:**

The purpose of this study was to objectively compare methodological approaches that might be utilized in designing an antimicrobial resistance (AMR) surveillance program in beef feedlot cattle. Specifically, four separate comparisons were made to investigate their potential impact on estimates for prevalence of AMR. These included investigating potential differences between 2 different susceptibility testing methods (broth microdilution and disc diffusion), between 2 different target bacteria (non-type-specific *E*. *coli* [NTSEC] and *Mannheimia haemolytica*), between 2 strategies for sampling feces (individual samples collected per rectum and pooled samples collected from the pen floor), and between 2 strategies for determining which cattle to sample (cattle that were culture-positive for *Mannheimia haemolytica* and those that were culture-negative).

**Results:**

Comparing two susceptibility testing methods demonstrated differences in the likelihood of detecting resistance between automated disk diffusion (BioMIC®) and broth microdilution (Sensititre®) for both *E*. *coli* and *M. haemolytica*. Differences were also detected when comparing resistance between two bacterial organisms within the same cattle; there was a higher likelihood of detecting resistance in *E*. *coli* than in *M. haemolytica*. Differences in resistance prevalence were not detected when using individual animal or composite pen sampling strategies. No differences in resistance prevalences were detected in *E*. *coli* recovered from cattle that were culture-positive for *M. haemolytica* compared to those that were culture-negative, suggesting that sampling strategies which targeted recovery of *E*. *coli* from *M. haemolytica*-positive cattle would not provide biased results.

**Conclusions:**

We found that for general purposes, the susceptibility test selected for AMR surveillance must be carefully chosen considering the purpose of the surveillance since the ability to detect resistance appears to vary between these tests depending upon the population where they are applied. Continued surveillance of AMR in *M. haemolytica* recovered by nasopharyngeal swab is recommended if monitoring an animal health pathogen is an objective of the surveillance program as results of surveillance using fecal *E*. *coli* cannot be extrapolated to this important respiratory pathogen. If surveillance of *E*. *coli* was pursued in the same population, study populations could target animals that were culture-positive for *M. haemolytica* without biasing estimates for AMR in *E*. *coli*. Composite pen-floor sampling or sampling of individuals per-rectum could possibly be used interchangeably for monitoring resistance in *E*. *coli*.

## Background

While several authors have suggested that there are questions that need to be addressed in order to optimize the structure of an antimicrobial resistance (AMR) surveillance program, many approaches have become accepted standard practices without investigating whether they are associated with differences in the ability to detect resistant isolates [1-3]. Scientists developing surveillance programs have had to rely on previous models of these systems or expert opinion when making decisions relative to the design. Unfortunately, this approach does not allow for evidence-based decision making when different design aspects have been based on suppositions that have not been formally investigated.

Decisions regarding antimicrobial susceptibility testing methodology and sampling approaches are crucial to obtaining relevant estimates of AMR in any population [4]. Disk diffusion and broth microdilution are the susceptibility tests commonly recommended for surveillance programs [3]. Broth microdilution is often considered the “surrogate” gold standard test for AMR when compared to disk diffusion. However, this opinion is partially based on application for clinical diagnostics rather than AMR surveillance, and the disk diffusion test is a less expensive option which may appeal to surveillance programs that are trying to optimize coverage relative to available funds [5-7]. Despite the putative superiority of broth microdilution over disk diffusion, it has not been rigorously investigated whether similar answers will be obtained using these tests to estimate resistance prevalence as they are applied for surveillance and other purposes. This information would help scientists make educated choices about the relative advantages and the comparability of data obtained regarding the apparent susceptibility of target isolates.

The bacterial agents that will be targeted by surveillance efforts must also be considered. The organisms monitored in AMR surveillance programs are often selected based on significance to public health and ease of sampling, yet relevance to the agriculture industry should also be considered in order to maintain support and to have practical relevance when designing a farm based surveillance program [8]. Specifically, in the beef feedlot industry, the resistance in *Escherichia coli* has often been assumed to be a reasonable surrogate for pathogens impacting cattle and people. However, feedlot cattle are not treated with antimicrobial drugs (AMDs) for this typically innocuous enteric organism. Rather, AMDs are most often used in feedlots to treat or prevent respiratory infections [8]. *Mannheimia haemolytica* is probably the most significant bacterial pathogen associated with respiratory disease syndromes in cattle and is therefore often the target organism when treating respiratory disease in this population [9]. Within the same cattle, it is unknown if resistance in *E*. *coli* is systematically different from that of *M. haemolytica*; therefore, using resistance in *E*. *coli* as a surrogate marker for resistance in *M. haemolytica* may not be appropriate or informative.

Another consideration when developing a farm based AMR surveillance program includes how to incorporate sampling strategies that minimize the inconvenience to the producer and that do not disturb typical operations [10]. Collecting fecal samples from individual cattle often requires restraining cattle in chutes and is therefore labor intensive, inconvenient, and can increase the risks for physical injury to cattle. A less invasive and generally more convenient approach would be to obtain samples of multiple fecal pats from the pen floors which could be mixed and cultured as a single composite sample that is representative of the whole pen. Pen floor sampling with pen level inferences has been successfully implemented in previous surveillance studies, yet there is little information about whether this type of sampling might provide different results compared to sampling individual samples in a surveillance program [11]. Improving our understanding of how the prevalence of AMR varies depending on whether samples are collected from individual cattle or from the pen floor would therefore be a valuable tool when considering the design of a surveillance program.

Conservation of financial and other resources is always a concern when developing surveillance programs, especially when programs span over long periods of time (i.e. years). Approaches to reduce costs should be considered carefully to ensure that there is no compromise to the validity of the collected data. For a surveillance program with a goal of comparing the resistances detected between 2 organisms (e.g. *E*. *coli* vs. *M. haemolytica*), the ability to make the comparison is limited by the lower of the two recovery rates. For example, *E*. *coli* is typically recovered from almost 100% of bovine fecal samples, but *M. haemolytica* typically can only be recovered from about 15% of live feedlot cattle using deep nasopharyngeal swabbing [12]. In a program where obtaining both organisms from one individual is required, resources could be spared by only testing *E*. *coli* isolated from cattle positive for *M. haemolytica*. While *E*. *coli* isolated from *M. haemolytica* negative cattle might contribute to power for analysis of *E*. *coli* resistance, these *E*. *coli* cannot be matched to a corresponding *M. haemolytica* isolate for analysis since none were recovered. However, if the manner of selecting only a subset of *E*. *coli* isolates (i.e. only those from *M. haemolytica* positive cattle) for testing creates any systematic bias, the results lose their validity.

The overall objective of this study was to explore several methodological approaches available for AMR surveillance in feedlot cattle to determine if they were associated with systematic differences in the likelihood of detecting resistance. Analyses were conducted to compare detected differences in resistance between two susceptibility testing methods, between two bacterial organisms, and between two sampling strategies. Additionally, an approach that might be used to identify subsets of samples to be cultured for *E*. *coli* relative to the culture status for *M. haemolytica* was evaluated for systematic bias.

## Methods

### Overview

Non-type specific *E*. *coli* (NTSEC) and *M. haemolytica* isolates were collected as part of a longitudinal project examining AMR in feedlot cattle (data not shown). Cattle were randomly enrolled in the project at the time of arrival at participating feedlots. Fecal samples were collected from pen floors and individual cattle and cultured for *E*. *coli*, while nasopharyngeal swabs were cultured for *M. haemolytica*. Subsets of isolates were purposively selected (described below) from the entire sample set and used in evaluations of whether systematic differences in resistance prevalences were associated with different methodological approaches. These comparisons included 1) susceptibility in the same isolates using broth microdilution or automated disk diffusion (for both NTSEC and *M. haemolytica*); 2) detection of resistance in NTSEC vs. *M. haemolytica* that were recovered from the same individuals; 3) detection of resistance in NTSEC recovered from fecal samples were obtained per rectum vs. isolates recovered from composite pen floor samples; 4) detection of resistance in NTSEC recovered from cattle that were culture-positive for *M. haemolytica* vs. those that were culture-negative for *M. haemolytica*.

### Study population

All cattle handling and sampling procedures were approved prior to the initiation of the study by the Animal Care Committee of the University of Calgary (Protocol Number M07031). Cattle sampled in this study were procured and managed at four western Canadian feedlots in south central Alberta under production conditions typical of those used at large commercial cattle feedlots throughout western Canada and the U.S. Participating feedlots had one-time holding capacities that ranged between 15,000 and 20,000 cattle, with pens housing capacities of 50-350 cattle. Commercial feedlots were purposively selected for participation based on their owners’ and managers’ willingness to participate, and the ability to collect data about exposures to antimicrobial drugs (AMDs) for individual cattle.

The cattle utilized in this study were procured through the auction market system from throughout Canada. Various cattle types were fed at these feedlots, including cattle of different entry weights, age classes (calves and yearlings), frame sizes, sources (e.g., ranch-direct and backgrounded cattle), and sexes (steers and heifers). Cattle entering these feedlots typically weighed about 225-400 kg, were managed in the feedlot for approximately 120-250 days, and were harvested when body weights reached 550-650 kg.

All cattle enrolled in the study were managed using the same standardized health and production procedures as per the protocols developed by specialists overseeing the health and production practices (FHMS). In brief, at arrival, each animal received a unique identification ear tag, a growth implant, vaccines against selected bacterial and viral pathogens, and a topical avermectin anthelmintic for parasite control. In cattle determined to have a greater than average risk of developing respiratory disease as determined using standardized risk profiles developed by FHMS health and production specialists, a parenteral antimicrobial drug was also administered at the time of arrival as a prophylactic or metaphylactic treatment. Water and feed were offered *ad libitum* throughout the feeding period; all diets were specially formulated to meet or exceed the National Research Council nutritional requirements for beef cattle [13]. The health of cattle was evaluated daily by trained feedlot personnel, and animals deemed to be sick were treated under the supervision of veterinarians using standardized protocols developed by FHMS.

### Sampling procedures

Cattle were enrolled in the study from 17 September 2007 to 16 January 2010. A two stage random sampling plan was used to determine which pens and individual cattle within those pens were selected for enrollment. The randomization scheme was applied throughout the study period, so the frequency of enrollment and sampling represented the frequency with which cattle enter feedlots in western Canada; predictably, higher volumes of cattle are placed in feedlots in the fall and lower volumes of cattle are placed in feedlots in the summer months. As cattle arrived at the feedlot, 30% of all newly formed pens of cattle were randomly selected for inclusion in the study using a randomization table. Within each selected pen, 10% of all cattle were enrolled at initial processing using a different, individual animal randomization table.

Enrolled individual cattle were sampled twice over the course of the study: during initial processing which occurred shortly after arrival to the feedlot (first sample time point) and later in the feeding period when cattle were rehandled for a variety of standard feedlot protocols (e.g., for replacement of growth-promoting implants) Standard protocols for group processing of cattle varied based upon the type of cattle being managed (i.e. calves vs. yearlings and ranch-direct vs. backgrounded calves) and can vary quite widely. As such, the timing for obtaining the second samples from cattle varied from 33-202 days on feed (DOF) with an average of 95.5 DOF (median = 80.0 DOF).

Two types of specimens were collected each time individual cattle were sampled: 1) a nasopharyngeal swab sample which was cultured for *M. haemolytica* and 2) a fecal sample collected per-rectum which was cultured for NTSEC. Nasopharyngeal samples were collected in the deep pharynx using a commercially available double guarded swab (# J273, Jorgensen Laboratories, Inc., Loveland, CO, USA). After collection, swabs were broken into a Cary Blair media tube (BBL CultureSwab™, CA90001-038, VWR International, Mississauga, Ontario). Fecal samples were collected per rectum using a new plastic palpation sleeve for every animal, and a minimum of 4 grams of feces was placed in a vial containing modified Cary Blair transport media (Enteric Transport Medium, 15 ml, Dalynn Biologicals Inc.) [14].

Pens were typically filled over several days, as cattle arrived at the feedlot on different trucks and as animals were processed and assigned to a pen. Composite fecal samples were collected from floors of pens allocated to the study soon after occupancy assignments were complete (first sample time point) and near the dates when individual animal samples were collected from animals assigned to that pen (second sample time point). Composite pen-floor samples were obtained using a new plastic spoon by placing approx. 0.5 to 1 g of feces that was collected from 20 fresh fecal pats into a new plastic container (minimum 10 g feces). The composite sample was mixed thoroughly, and approximately 4 g of feces from each container was then transferred into a vial containing modified Cary Blair transport media (Enteric Transport Medium, 15 ml, Cat#F01W, Dalynn Biologicals Inc., Calgary, Alberta) [14].

*Sample Transport and Data Storage*--All samples (nasopharyngeal swabs, rectal feces, and composite pen-floor feces) were labeled with the date and the pen number (and the individual ID for individual cattle samples), refrigerated in a chilled cooler and transported to the microbiology laboratory (Agriculture and Agri-Food Canada Lethbridge Research Station, Lethbridge, Alberta) by overnight courier.

### Laboratory procedures

Upon arrival at the lab, fecal samples were kept refrigerated at 4°C until nasopharyngeal samples were processed. As a standard protocol for this surveillance project, fecal samples collected per rectum from individual cattle were only processed to recover NTSEC isolates if *M. haemolytica* was cultured from that animal’s nasopharyngeal swab. This approach was used to conserve resources while still allowing comparison of resistance outcomes of both NTSEC and *M. haemolytica* obtained from the same cattle. However, in order to evaluate whether bias was introduced by preferentially culturing feces from *M. haemolytica* positive-cattle, 225 *M. haemolytica*-negative cattle were purposively selected and NTSEC were cultured from fecal samples collected per rectum. All composite pen-floor fecal samples were cultured and to recover NTSEC regardless of the *M. haemolytica* status of cattle in the pen.

### Nasopharyngeal swabs

Nasal swabs were aseptically removed from transport vials and the tips were vortexed in a centrifuge tube at high speed for 30 seconds and then allowed to sit undisturbed for at least 10 minutes. Samples of the suspension recovered from swabs (100 μl) were spread onto blood agar containing 15 μg/mL bacitracin (BAC plates) and incubated overnight at 37°C. Additional plates were also inoculated with *M. haemolytica* ATCC strain 33396 and *M*. *glucosida* ATCC strain 38457 as positive controls. Three to five colonies with morphology typical of *M. haemolytica* (round, medium sized, moist, white-grey colored colonies with some degree of hemolysis evident) were selected for further analysis, streaked onto BAC plates and incubated for 8-12 hrs at 37°C. Isolated colonies were evaluated to confirm purity and to verify that the morphology was similar to the reference plate. Isolates that were oxidase and catalase positive were preliminarily identified as *M. haemolytica* and were stored in 20% glycerol stocks at -80°C until further phenotypic and genotypic characterization was conducted. After thawing, phenotypic tests were performed using Rosco diagnostic tablets (Diatabs®), including alpha-fucosidase, beta-galactosidase, beta-glucosidase, beta-xylosidase, D-xylose, esculin hydrolysis, indole, L-arabinose, maltose, mannitol, ornithine decarboxylase, sorbitol, trehalose, and urease [15]. A multiplex PCR assay was used as the final confirmation for *M. haemolytica* isolates and all confirmed isolates were tested for antimicrobial susceptibility [16]. Reference strains of *M. haemolytica* and *M*. *glucosida* as well as negative controls were included in evaluations as references for comparison.

### Composite and individual fecal samples

Fecal samples were processed by mixing the Cary-Blair transport medium to create uniform slurry [14]. A sterile cotton swab was used to streak samples onto MacConkey agar (MAC) and incubated for 24 hours at 37°C. Isolates that fermented lactose and had appropriate colony morphology were subcultured on lysogeny broth (LB), incubated at 37°C overnight and then tested for indole. A presumptive identification of NTSEC was based on colony morphology, lactose fermentation and positive indole reaction. Up to three NTSEC colonies were selected from each individual animal’s fecal sample as were five colonies from each composite fecal sample, and isolates were archived for susceptibility testing by freezing at -80°C in 30% glycerol.

### Antimicrobial susceptibility testing

All NTSEC and *M. haemolytica* isolates included in these analyses were tested for susceptibility to standardized panels of AMDs by disk diffusion and broth microdilution (Sensititre® panel type: CMV1AGNF). Both procedures were conducted according to rigorously standardized protocols as proscribed by the Clinical Laboratory Standards Institute [17]. The AMDs included on both the disk diffusion and broth microdilution susceptibility panels for NTSEC and *M. haemolytica* are listed in Tables 1, 2, 3 and 4. The antimicrobial drug panels were developed independently for surveillance purposes and as such AMDs included on the 2 panels were not identical. Results of disk diffusion testing were recorded as zone diameters as determined using an automated zone measurement system (BioMIC®), and results of broth microdilution testing were recorded as the minimum inhibitory concentration (MIC). *Escherichia coli* ATCC 25922 and *Streptococcus pneumoniae* ATCC 49619 were used in quality control assessments for both susceptibility testing procedures. Additionally, *Staphylococcus aureus* ATCC 29213 was used for quality control in the broth microdilution testing while *Staphylococcus aureus* ATCC 25923 was used in the disk diffusion testing procedures.

**Table 1:** **Interpretive criteria for *E coli *** **using broth microdilution susceptibility testing reported as minimum inhibitory concentrations (μg/ml)**

**Antimicrobial**	**Susceptible**	**Intermediate**	**Resistant**	**Reference**
Amikacin	≤16	32	≥64	CLSI M100-S22, 2012
Ampicillin	≤8	16	≥32	CLSI M100-S22, 2012
Amoxicillin-Clavulanate	≤8/4	16/8	≥32/16	CLSI M100-S22, 2012
Cefoxitin	≤8	16	≥32	CLSI M100-S22, 2012
Ceftiofur	≤2	4	≥8	CLSI M31-A4, 2013
Ceftriaxone	≤1	2	≥4	CLSI M100-S22, 2012
Chloramphenicol	≤8	16	≥32	CLSI M100-S22, 2012
Ciprofloxacin	≤1	2	≥4	CLSI M100-S21, 2011
Gentamicin	≤4	8	≥16	CLSI M100-S22, 2012
Kanamycin	≤16	32	≥64	CLSI M100-S22, 2012
Nalidixic Acid	≤16	-	≥32	CLSI M100-S22, 2012
Streptomycin	≤32	-	≥64	NARMS Executive Report 2009
Sulfisoxazole	≤256	-	≥512	CLSI M100-S22, 2012
Tetracycline	≤4	8	≥16	CLSI M100-S22, 2012
Trimethoprim-Sulfamethoxazole	≤2/38	-	≥4/76	CLSI M100-S22, 2012

*CLSI* = Clinical and Laboratory Standards Institute.

**Table 2:** **Interpretive criteria for *M haemolytica *** **using broth microdilution susceptibility testing reported as minimum inhibitory concentrations (μg/ml)**

**Antimicrobial**	**Susceptible**	**Intermediate**	**Resistant**	**Reference**
Amikacin	≤16	32	≥64	*CLSI M100-S22, 2012
Ampicillin	≤0.5	-	-	CLSI M45-A2, 2010
Amoxicillin-Clavulanate	≤0.5/0.25	-	-	CLSI M45-A2, 2010
Cefoxitin	≤8	16	≥32	*CLSI M100-S22, 2012
Ceftiofur	≤2	4	≥8	CSLI M31-A4, 2013
Ceftriaxone	≤1	2	≥4	*CLSI M100-S22, 2012
Chloramphenicol	≤8	16	≥32	*CLSI M100-S22, 2012
Ciprofloxacin	≤1	2	≥4	*CLSI M100-S21, 2011
Gentamicin	≤4	8	≥16	*CLSI M100-S22, 2012
Kanamycin	≤16	32	≥64	*CLSI M100-S22, 2012
Nalidixic Acid	≤16	-	≥32	*CLSI M100-S22, 2012
Streptomycin	≤32	-	≥64	NARMS Executive Report 2009
Sulfisoxazole	≤256	-	≥512	*CLSI M100-S22, 2012
Tetracycline	≤2	4	≥8	CLSI M31-A4, 2013
Trimethoprim-Sulfamethoxazole	≤0.5/9.5	-	-	CLSI M45-A2, 2010

*Interpretive criteria for *E*. *coli* used in lieu of *M. haemolytica* since *CLSI* does not define breakpoints for *M. haemolytica* and these antimicrobial drugs.

*CLSI* = Clinical and Laboratory Standards Institute.

**Table 3:** **Interpretive criteria for *E coli *** **using disk diffusion susceptibility testing reported as inhibition zone diameters (mm)**

**Antimicrobial**	**[Disk] (μg)**	**Susceptible**	**Intermediate**	**Resistant**	**Reference**
Ampicillin	10	≥17	14-16	≤13	CLSI M100-S22, 2012
Amoxicillin- Clavulanate	20/10	≥18	14-17	≤13	CLSI M100-S22, 2012
Ceftazidine	30	≥18	15-17	≤14	CLSI M100-S18, 2008
Ceftiofur	30	≥21	18-20	≤17	CLSI M31-A4, 2013
Enrofloxacin	5	≥21	17-20	≤16	*CLSI M31-A4, 2013
Florfenicol	30	≥19	15-18	≤14	*CLSI M31- A4, 2013
Neomycin	30	≥17	-	≤12	*CLSI M31-A3, 2008
Streptomycin	10	≥15	12-14	≤11	CLSI M100-S18, 2008
Sulfisoxazole	300	≥17	13-16	≤12	CLSI M100-S22, 2012
Tetracycline	30	≥15	12-14	≤11	CLSI M31-A4, 2013
Trimethoprim-Sulfamethoxazole	1.25/23.75	≥16	11-15	≤10	CLSI M100-S22, 2012

*Interpretive criteria for *M. haemolytica* used in lieu of *E*. *coli* since *CLSI* does not define breakpoints for *E*. *coli* in cattle for these antimicrobial drugs.

*CLSI* = Clinical and Laboratory Standards Institute.

**Table 4:** **Interpretive criteria for *M haemolytica *** **using disk diffusion susceptibility testing reported as inhibition zone diameters in (mm)**

**Antimicrobial**	**[Disk] (μg)**	**Susceptible**	**Intermediate**	**Resistant**	**Reference**
Ampicillin	10	≥27	-	-	CLSI M45-A2, 2010
Amoxicillin- Clavulanate	20/10	≥27	-	-	CLSI M45-A2, 2010
Ceftiofur	30	≥21	18-20	≤17	CLSI M31-A3, 2008
Danofloxacin	5	≥22	-	-	CLSI M31-A3, 2008
Enrofloxacin	5	≥21	17-20	≤16	CLSI M31-A4, 2013
Florfenicol	30	≥19	15-18	≤14	CLSI M31-A4, 2013
Gentamicin	10	≥15	-	≤12	Catry et al., 2007
Spectinomycin	100	≥14	11-13	≤10	CLSI M31-A3, 2008
Sulfisoxazole	300	≥17	13-16	≤12	CLSI M31-A3, 2008
Tetracycline	30	≥23	-	-	CLSI M45-A2, 2010
Tilmicosin	15	≥14	11-13	≤10	CLSI M31-A4, 2013
Trimethoprim-Sulfamethoxazole	1.25/23.75	≥24	-	-	CLSI M45-A2, 2010
Tulathromycin	30	≥18	15-17	≤14	CLSI M31-A4, 2013

*CLSI* = Clinical and Laboratory Standards Institute.

### Interpretive criteria

Non-type specific *E*. *coli* and *M. haemolytica* isolates were categorized as susceptible (S), intermediate (I), or resistant (R) to antimicrobials using interpretive criteria published by the Clinical and Laboratory Standards Institute (CLSI) where available (Tables 1, 2, 3 and 4) [17-21]. However, the CLSI does not have published interpretive criteria for cattle regarding all of the drugs that were evaluated in this study. When possible, interpretive criteria that were specific for cattle and the test method/drug/bacteria combination were used (e.g., breakpoints for broth microdilution evaluating ceftiofur and *M. haemolytica*). When these were not available, breakpoints for cattle regarding the drug and other bacterial species were used. If these were not available, then interpretive criteria published for humans regarding bacteria-drug combinations were used. The exceptions to this approach were that breakpoints for broth microdilution testing of NTSEC for streptomycin and disk diffusion testing of *M. haemolytica* for gentamicin. Streptomycin breakpoints were based on criteria used by the National Antimicrobial Resistance Monitoring System of the USDA and FDA [22], which were established using MIC distributions for bacteria and the presence of known resistance genes or mutations. Gentamicin interpretive criteria were based on a published evaluation [23].

### Analysis

To facilitate analysis involving logistic regression, isolate susceptibility was dichotomized as resistant and non-resistant (which included both intermediate and susceptible classifications). For AMDs that did not have a published intermediate susceptibility classification (i.e., published reference only include values for susceptible classification of isolates [e.g., ampicillin for *M. haemolytica*]), any isolate not classified as susceptible was categorized as being resistant. The outcome for logistic models was the phenotypic resistance or non-resistance of each NTSEC or *M. haemolytica* isolate, and separate models were evaluated in parallel for each antimicrobial drug tested. Analysis of the variance structure relative to the hierarchy of the data (feedlot-pen-animal-isolate) revealed that almost all of the clustering (correlation in resistance outcomes) occurred at the isolate level (data not shown, MLwiN version 2.02, Center for Multilevel Modeling, University of Bristol). As such, lack of independence among isolates was controlled in models using generalized estimating equations (GEE) using an exchangeable correlation structure. Separate models for each antimicrobial drug and for each methodological comparison were built using *a priori* understandings of the hierarchical data structure using commercially available software (SAS version 9.2, SAS Institute Inc., Cary, NC). Odds ratios, 95% confidence intervals (95%CI), and the associated *P*-values were obtained for each model. Due to lack of precision in the estimates and the impact on model stability, models were not evaluated if resistance to a particular antimicrobial was less than 2%.

### Isolates and data analyses

### Susceptibility test comparison

Isolates evaluated by both susceptibility testing methods (disk diffusion and broth microdilution) were included for the comparison of detected differences in resistance between two susceptibility tests. Conditional logistic regression with matching on the isolate level was used to evaluate the association between resistance to each AMD and susceptibility test. The type of susceptibility test was the only predictor included in these models (automated disk diffusion vs. broth microdilution). There were 7 AMDs that could be analyzed in these models as these drugs were evaluated on both susceptibility panels (ampicillin, amoxicillin-clavulanic acid, ceftiofur, streptomycin, sulfisoxazole, tetracycline, and trimethoprim-sulfamethoxazole). Separate models were analyzed for NTSEC recovered from individuals and all *M. haemolytica* isolates.

### Organism comparison

Isolates of NTSEC and *M. haemolytica* that were recovered from the same individuals were evaluated in conditional logistic regression models (matching on isolate) to investigate systematic differences in resistance for the two organisms. Only susceptibility results from testing with broth microdilution were included in these analyses. Separate models were evaluated for each resistance outcome (i.e., drug) and organism was the only predictor included in these models (NTSEC vs. *M. haemolytica*).

### Sampling strategy comparison for NTSEC

To investigate the potential that there may be differences in resistance prevalences among NTSEC recovered from individual fecal samples when compared to those recovered from composite (pen-floor) samples, isolates recovered from these two sample types were compared using logistic regression. Only susceptibility results from testing with broth microdilution were included in these analyses. Lack of independence among isolates was controlled in models using generalized estimating equations (GEE) using an exchangeable correlation structure. Separate models were evaluated for each AMD and the sample type was the only predictor included in these models (fecal samples collected per rectum from individuals vs. composite fecal samples collected from pen floors). Since pens were occasionally stocked over a number of days, only the isolates that were recovered from composite and individual samples that had been collected on the same day were included in these analyses.

### Selective testing of NTSEC isolates

Logistic regression was used to compare the likelihood for detecting resistance prevalences among NTSEC recovered from cattle that were culture-positive for *M. haemolytica* versus those that were culture-negative. Only susceptibility results from testing with broth microdilution were included in these analyses. Only isolates collected from culture-positive and culture-negative cattle that were housed in the same pens and sampled within the same time frame were included in these analyses. Lack of independence among isolates recovered from individuals housed within the same pens was controlled using GEE using an exchangeable correlation structure, and separate models were investigated for each AMD. The *M. haemolytica* culture status of individuals (culture-positive vs. culture-negative) was included as the fixed effect of interest, and additional predictor variables were controlled in the analyses to account for potential differences in resistance that might be associated with feedlot, sampling time-frame (i.e., first or second sample), and a single interaction term between sample time-frame and *M. haemolytica* culture status.

## Results

### Antimicrobial susceptibility test comparison

A total of 3362 NTSEC isolates recovered from individual fecal samples and 1574 *M. haemolytica* nasopharyngeal isolates were evaluated for susceptibility using both broth microdilution and disk diffusion. Results indicated that there were strong differences in the likelihood of resistance classification for NTSEC isolates from individual fecal samples for 5 of 6 AMDs (Table 5). Automated disk diffusion was more likely than broth microdilution to classify the isolates as resistant to ampicillin, to ceftiofur, to streptomycin, and to trimethoprim-sulfamethoxazole. However, disk diffusion was less likely to classify NTSEC isolated from individual samples as tetracycline resistant than broth microdilution. The model for amoxicillin-clavulanic acid would not converge, presumably because of the low prevalence of resistance (<2%).

**Table 5:** **Likelihood of identifying resistance in isolates of NTSEC or *M Haemolytica *** **when tested using disk diffusion versus broth microdilution**

**Resistance outcome**	**Susceptibility testing method**	**NTSEC (n=3362)**			***M. haemolytica *** **(n=1574)**		
		**OR**^**1**^	**95% CI**^**2**^	***P*-value**	**OR**^**1**^	**95% CI**^**2**^	***P*-value**
Ampicillin	DD^3^	6.3	2.9 – 14.3	<0.0001	20.0	5.0 – 100	<0.0001
	BM^4^	ref			ref		
Amoxicillin-Clavulanate	DD	−^5^	–	–	10.0	3.0 – 33.3	0.0001
	BM				ref		
Ceftiofur	DD	5.6	1.2 – 25.0	0.03	−^5^	–	–
	BM	ref					
Streptomycin	DD	2.0	1.6 – 2.7	<0.0001	−^5^	–	–
	BM	ref					
Sulfisoxazole	DD	1.3	0.9 – 1.9	0.11	−^5^	–	–
	BM	ref					
Tetracycline	DD	0.6	0.5 – 0.8	0.0003	25.0	7.7 – 100	<0.0001
	BM	ref			ref		
TMS^6^	DD	4.8	2.0 – 11.1	0.0004	−^5^	–	–
	BM	ref					

^1^Odds ratio comparing disk diffusion to broth microdilution as the reference category.

^2^95% confidence intervals determined using Wald statistics.

^3^Disk Diffusion.

^4^Broth Microdilution.

^5^Model was not stable because of low resistance prevalence.

^6^Trimethoprim-Sulfamethoxazole.

There were also strong differences in the likelihood of detecting resistance for *M. haemolytica*, where automated disk diffusion was more likely than broth microdilution to classify resistance to ampicillin, amoxicillin-clavulanic acid, and tetracycline. Models for ceftiofur, streptomycin, sulfisoxazole, and trimethoprim-sulfamethoxazole did not converge, presumably because of the low prevalence of resistance (all were <2%).

### Organism comparison

Isolates of NTSEC and *M. haemolytica* that were both recovered from the same 2190 individuals were identified for this comparison and evaluated by broth microdilution. Results indicate that there was a strong statistically detectable difference in the likelihood of detecting resistance in NTSEC for all drugs except nalidixic acid, when compared to *M. haemolytica* (Table 6). Non-type specific *E*. *coli* was much more likely to be classified as resistant to ampicillin, streptomycin, sulfisoxazole, tetracycline, and trimethoprim-sulfamethoxazole, but *M. haemolytica* was more likely than NTSEC to be classified as resistant to kanamycin. Resistance in other drugs could not be compared in these analyses because of low resistance prevalence which created instability in regression models (amikacin, amoxicillin-clavulanate, cefoxitin, ceftiofur, ceftriaxone, chloramphenicol, ciprofloxacin, gentamicin, and nalidixic acid).

**Table 6:** **Likelihood of identifying resistance among paired isolates of NTSEC and *M haemolytica *** **that were recovered from the same individuals on the same day (n=2190)**^**1**^

**Resistance outcome**^**2**^	**Organism**	**OR**^**3**^	**95% CI**^**4**^	***P-*value**
Ampicillin	NTSEC	3.1	2.0 - 4.8	<0.0001
	*M. haemolytica*	Reference		
Kanamycin	NTSEC	0.2	0.1 - 0.3	<0.0001
	*M. haemolytica*	Reference		
Nalidixic acid	NTSEC	4.0	0.8 - 20.0	0.08
	*M. haemolytica*	Reference		
Streptomycin	NTSEC	3.3	3.3 - 10.7	<0.0001
	*M. haemolytica*	Reference		
Sulfisoxazole	NTSEC	50.0	25.0 - 100.0	<0.0001
	*M. haemolytica*	Reference		
Tetracycline	NTSEC	33.3	50.0 - 100.0	<0.0001
	*M. haemolytica*	Reference		
Trimethoprim- Sulfamethoxazole	NTSEC	11.1	2.7 - 50.0	0.001
	*M. haemolytica*	Reference		

^1^Susceptibility testing was performed using broth microdilution.

^2^Other drugs could not be analyzed because of low resistance prevalence (amikacin, amoxicillin-clavulanate, cefoxitin, ceftiofur, ceftriaxone, chloramphenicol, ciprofloxacin, gentamicin, and nalidixic acid).

^3^Odds ratio.

^4^95% confidence intervals determined using Wald statistics.

### Sampling strategy comparison

A total of 412 NTSEC recovered from 137 individual fecal samples collected per rectum on the second sampling day were used in this analysis, along with 198 NTSEC isolates collected from 40 composite pen-floor samples that were collected from the same pens on the same days. Controlling for the lack of independence created by analyzing multiple isolates from the same sample and obtaining multiple samples from cattle housed in the same pen, the proportion of resistant isolates was not different for any of the drugs evaluated (ampicillin, chloramphenicol, kanamycin, nalidixic acid, streptomycin, sulfisoxazole, tetracycline, and trimethoprim-sulfamethoxazole; *P*>0.30; Table 7). Resistance in other drugs could not be compared in these analyses because of low resistance prevalence which created instability in regression models (amikacin, amoxicillin-clavulanate, cefoxitin, ceftiofur, ceftriaxone, ciprofloxacin, gentamicin, and nalidixic acid).

**Table 7:** **Likelihood of identifying resistance among NTSEC isolates cultured from feces collected as composite pen floor samples or as individual samples collected per rectum on the same sampling days**^**1**,**2**^

**Resistance outcome**^**3**^	**Type of fecal sample**	**OR**^**4**^	**95% CI**^**5**^	***P-*value**
Ampicillin	Individual	0.8	0.3 – 1.8	0.58
	Composite	Reference		
Chloramphenicol	Individual	0.9	0.4 – 2.5	0.94
	Composite	Reference		
Kanamycin	Individual	0.6	0.2 – 2.5	0.5
	Composite	Reference		
Nalidixic Acid	Individual	3.5	0.3 – 44.8	0.41
	Composite	Reference		
Streptomycin	Individual	1.3	0.8 – 2.2	0.31
	Composite	Reference		
Sulfisoxazole	Individual	1.2	0.7 – 2.2	0.5
	Composite	Reference		
Tetracycline	Individual	0.9	0.6 – 1.7	0.92
	Composite	Reference		
Trimethoprim- Sulfamethoxazole	Individual	1.7	0.4 – 8.5	0.5
	Composite	Reference		

^1^n=412 isolates recovered from 137 fecal samples collected from individual cattle, and n=198 isolates recovered from 40 composite pen floor fecal samples.

^2^Susceptibility testing was performed using broth microdilution.

^3^Other drugs could not be analyzed because of low resistance prevalence (amikacin, amoxicillin-clavulanate, cefoxitin, ceftiofur, ceftriaxone, ciprofloxacin, gentamicin, and nalidixic acid).

^4^ Odds Ratio. Analyses controlled for potential lack of independence related to repeated measures and hierarchical data structure using generalized estimating equations.

^5^95% confidence intervals.

### Selective testing of E. Coli isolates

The NSTEC isolates used in this comparison were recovered from a total of 186 cattle (n=377 isolates) that were culture-positive for *M. haemolytica* and 77 that were culture-negative (n=225 isolates). Modeling as described, results indicated that there was no detectable difference in the proportion of resistant isolates when comparing NTSEC isolates recovered from these 2 groups (Table 8). Resistance in other drugs could not be compared in these analyses because of low resistance prevalence which created instability in regression models (amikacin, amoxicillin-clavulanate, cefoxitin, ceftiofur, ceftriaxone, chloramphenicol, ciprofloxacin, gentamicin, kanamycin, nalidixic acid, trimethoprim-sulfamethoxazole).

**Table 8:** **Likelihood of identifying resistance among NTSEC isolates recovered from individuals that were culture**-**positive for *M haemolytica *** **to those that were culture-negative, stratified by sample time**^**1,2**^

**Resistance outcome in fecal *E*. *coli***^**3**^	**Sample Time**	***M. haemolytica *** **Culture Status**^**4**^	**OR**^**5**^	**95% CI**^**6**^	***P-value***
Ampicillin	First	Positive	1.2	0.3 – 4.8	0.78
		Negative	ref		
	Second	Positive	3.3	0.4 – 26.8	0.19
		Negative	ref		
Streptomycin	First	Positive	0.4	0.2 – 0.9	0.04
		Negative	ref		
	Second	Positive	0.5	0.1 – 2.3	0.46
		Negative	ref		
Sulfisoxazole	First	Positive	0.8	0.4 – 1.7	0.56
		Negative	ref		
	Second	Positive	1.5	0.5 – 4.4	0.50
		Negative	ref		
Teteracyline	First	Positive	1.1	0.5 – 2.5	0.82
		Negative	ref		
	Second	Positive	0.9	0.4 – 2.2	0.90
		Negative	ref		

^1^n=377 isolates recovered from 186 individuals that were culture-positive for *MH*, and n=225 isolates recovered from 77 individuals that were culture-negative for *MH*.

^2^Susceptibility testing was performed using broth microdilution.

^3^Other drugs could not be analyzed because of low resistance prevalence (amikacin, amoxicillin-clavulanate, cefoxitin, ceftiofur, ceftriaxone, chloramphenicol, ciprofloxacin, gentamicin, kanamycin, nalidixic acid, trimethoprim-sulfamethoxazole).

^4^Culture status regarding recovery of *M. haemolytica* from nasopharyngeal swabs.

^5^Analyses controlled for repeated measures and hierarchical data structure using generalized estimating equations (GEE), and fixed effects for feedlot, sample time, *M. haemolytica* culture-status, and a single interaction term for sample time*M. haemolytica culture-status.

^6^95% confidence intervals.

## Discussion

This study was performed because of the practical questions that naturally arose when attempting to design a large-scale surveillance program for AMR in beef feedlot cattle. Specifically these questions were: Does it matter which susceptibility testing methods are used? Is resistance different for enteric surveillance targets compared to testing of a respiratory pathogen? How should fecal samples be collected? And, does selective sampling of cattle bias results? These results suggest the method for evaluating susceptibility and the target bacteria chosen for surveillance programs must be considered carefully relative to the purpose for conducting surveillance, as these choices will systematically affect the results of the investigations. However, there was no difference in resistance estimates obtained from fecal samples collected per rectum or as composite pen floor samples, nor did *M. haemolytica* culture status affect results related to resistance in NTSEC. Thus surveillance programs could be flexible relative to these factors.

As with all work involving *in vitro* test results for antimicrobial resistance, analyses were heavily dependent upon interpretive criteria used in classifying susceptibility of isolates. Ideally, breakpoints specific to animal species, disease, pathogen, drug, and the administration regimen (dose, route, duration, and frequency) should be used for the most clinically valid interpretations. However, approved guidelines have not been defined for all of the antimicrobial drugs tested in this study regarding feedlot cattle. Therefore, each antimicrobial drug was carefully investigated relative to NTSEC and *M. haemolytica* to identify appropriate CLSI interpretive criteria when possible and published acceptable alternatives when CLSI criteria were not defined (Tables 1, 2, 3 and 4). The biological differences between these organisms likely contribute to whether each is resistant (or not) to specified concentrations of antimicrobial drug. Therefore, the breakpoint differences relative to the organism may affect or even bias the results of these comparisons. The very large differences in the proportion of resistant isolates when comparing the two target organisms likely represent real differences, but the magnitude of these differences might change if different breakpoints were used (such as epidemiological breakpoints) [24].

A low prevalence of resistance to many of drugs evaluated in this study has been previously described in NTSEC and *M. haemolytica* recovered from feedlot cattle and therefore was not unexpected [11,12,25,26]. However, this low resistance prevalence limited the ability to make inferences regarding our research questions with all of the drugs that were evaluated. Regardless, given the diversity of drugs that were included these analyses and the low prevalence that has been documented in multiple studies, it seems unlikely that contradictory findings would be obtained if we had been able to include these drugs in the regression modeling.

While there were differences noted by directly comparing the detection of resistance with broth microdilution or disk diffusion, these observations cannot be extrapolated to provide unbiased estimates of test accuracy (sensitivity and specificity), as neither can be considered a perfect reference. Therefore, while these results showed that for most of the drugs evaluated there were strong differences in the likelihood classifying organisms as being resistant, the superiority of one test over the other for correct classification of resistance cannot be asserted. Again, the interpretive criteria used in this study could have affected the estimates for resistance classification. While the interpretive criteria established by CLSI are intended to provide the same classification results for broth microdilution and disk diffusion when comparing test results for the same isolates and extreme care was used to ensure that appropriate breakpoints were used throughout this investigation, results could have been affected by the absence of standardized criteria for all bacteria-drug combinations and specifically defined for cattle. Other studies have evaluated the MICs estimated by algorithms based on the zone diameters and compared them to the MICs determined by broth microdilution to show both close agreement as well as widely divergent results [27,28]. Interestingly, results regarding tetracycline resistance indicated that broth microdilution was more likely to classify tetracycline resistance in NTSEC whereas disk diffusion was more likely to classify tetracycline resistance in *M. haemolytica*. These differences could partially be attributed to the previously discussed biological differences between the organisms as well as the detection differences between the susceptibility tests.

Investigations of AMR in animal populations have much greater relevance to producers if important bacterial pathogens are incorporated in surveillance efforts alongside monitoring an indicator of AMR for the purposes of public health surveillance. However, based on previous research, it was known that the *M. haemolytica* would not be recoverable from all cattle, while NTSEC is ubiquitous [12]. Therefore, as a measure to aid efficiency and cost-effectiveness in the larger surveillance program within which these investigations were nested, it was decided to only recover NTSEC from the individuals that were culture-positive for *M. haemolytica*. The ability to recover *M. haemolytica* might be related to the treatment history of an individual as well as the sample time (arrival vs. later in the feeding period). Further, the ability to recover this bacterium could also be related to its resistance status. Therefore, a direct comparison between *M. haemolytica* positive and *M. haemolytica* negative NTSEC isolates was warranted to verify if results might be biased by the design consideration to only recover NTSEC from individuals culture positive for *M*. *haemoltyica*. Other factors were also controlled in these analyses which may have affected the likelihood of detecting resistant NTSEC. Even though the interaction (sample time*M. haemolytica status) was not found to be statistically significant in any of the models, it was still forced into models due to its biological relevance.

There was wide variability in the timing of when the second samples were collected because this sampling was coordinated with the need to restrain and handle individual cattle for routine management procedures (i.e., re-implanting) This routine handling occurs at different times because of variability in cattle weights/ages and sex, in addition to the practical logistics of managing large feedlots. Regardless, this variability should not have systematically altered results of this study as comparisons of multiple bacterial isolates recovered from individual cattle were limited to those recovered on the same day (i.e., fecal and nasopharyngeal samples were collected on the same day). Also, for comparisons of NSTEC isolates recovered from cattle that were culture-positive or culture-negative for *M. haemolytica*, the distributions of sampling days (DOF) were similar and sampling time was included as a variable in the analysis.

## Conclusions

We found that for use in AMR surveillance, the susceptibility test must be carefully chosen considering the purpose of the surveillance since the ability to correctly classify target organisms apparently can vary between these tests. This study was not designed to evaluate the accuracy of either test, but this investigation demonstrates that results for the two tests varied among the different drugs. Surveillance of AMR in *M. haemolytica* recovered from feedlot cattle is appropriate in order to improve the relevance by inclusion of an animal health pathogen, especially since AMR in NTSEC does not predict results for *M. haemolytica*. Sampling strategies used in AMR surveillance for NTSEC recovered from feedlot cattle could possibly employ composite pen floor fecal sampling interchangeably with sampling feces from individual cattle per rectum. Feces could be sampled from individuals to recover NTSEC without regard for *M. haemolytica* culture status as an efficiency when both agents are targets for surveillance.

## Abbreviations

AMR: Antimicrobial resistance; AMDs: Antimicrobial drugs; NTSEC: Non-type specific *E*. *coli*; Amox-Clav: Amoxicillin-clavulanic acid; Trimeth-Sulfa: Trimethoprim-sulfamethoxazole.

## Competing interests

The authors declare that they have no competing interests related to this manuscript.

## Authors’ contributions

KMB participated in study design, conducted all statistical analyses, participated in interpretation of analyses, and drafted the manuscript. SPG and TAM participated in study design, provided oversight of laboratory analysis, and participated in interpretation of analyses. SC participated in study design and assisted with interpretation of analyses. CWB participated in study design, provided oversight for sample collection, and assisted with interpretation of analyses. PSM conceived of the study, participated in its design and coordination and helped to draft the manuscript. All authors have read and approved the final manuscript.
